# Continuous Meniscal Repair Technique Allows for Shorter Operative Time and Learning Curve Compared With Traditional Vertical Mattress Technique in Controlled Arthroscopic Training in Porcine Model

**DOI:** 10.1016/j.asmr.2024.100957

**Published:** 2024-06-10

**Authors:** José Leonardo Rocha de Faria, João Vieira de Almeida Neto, Bruno Couto Gonçalves, Douglas Mello Pavão, João Antonio Matheus Guimarães, Valdeci Manoel de Oliveira, Robert F. LaPrade, Djalma Rabelo Ricardo, Rodrigo Salim

**Affiliations:** a Knee Surgery Center, National Institute of Traumatology and Orthopedics (INTO), Rio de Janeiro, RJ, Brazil; b School of Medicine from University of São Paulo–USP Ribeirão Preto, SP, Brazil; c Therezinha de Jesus Hospital and Maternity, Juiz de Fora, MG, Brazil; d Regional Hospital of the North Coast, Caraguatatuba, SP, Brazil; e SUPREMA—Faculty of Medical and Health Sciences of Juiz de For a, Juiz de Fora, MG, Brazil; f General Military Hospital of Juiz de Fora, Juiz de Fora, MG, Brazil; g Research Division, National Institute of Traumatology and Orthopedics, Rio de Janeiro, RJ, Brazil; h Twin Cities Orthopaedics, Edina, Minnesota, U.S.A.; i University of Minnesota Medical School, Minneapolis, Minnesota, U.S.A.

## Abstract

**Purpose:**

To compare the amount of time used to perform meniscal suturing on a standardized lesion using either a traditional or continuous arthroscopic suturing technique.

**Methods:**

A preclinical study was carried out with 21 medical doctors who underwent training in the 2 modalities of meniscal repair by arthroscopy in an animal model laboratory. Participants performed both types of sutures with a previously standardized lesion. The execution time of the techniques was measured, and an experienced surgeon evaluated the stability of a meniscal tear after the repair. Data were analyzed using a *t* test for paired samples to calculate the difference between the execution times of the techniques.

**Results:**

The time required to perform the continuous meniscal suture was shorter than that of the traditional suture. After statistical analysis, the time difference between the techniques was significant (mean difference 4:17 ± 5:30 minutes; 95% confidence interval, 1:46–6:46 minutes). Surgeons took less time than residents for the traditional suture (*P* = .036), but the times were similar for the continuous suture. This suggests that experience level has a greater effect on the time needed for the traditional suture than for the continuous suture.

**Conclusions:**

The continuous suture technique was performed in a shorter time compared with the traditional suture technique in a porcine model.

**Clinical Relevance:**

The results of this preclinical study suggest that the continuous vertical inside-out meniscal suture technique can enhance surgical procedures for longitudinal tears requiring ≥4 stitches, offering a faster and more intuitive learning curve compared with the traditional inside-out suture technique.

Studies have shown that meniscal repair is beneficial.^1^^,^^2^ However, meniscal suturing requires training and arthroscopic skill, even for more experienced surgeons.^3^, ^4^, ^5^, ^6^ The technique considered the gold standard for performing meniscal repairs is the traditional inside-out (IO) technique.^7^^,^^8^

A continuous meniscal suturing technique has been described that potentially appears to be faster and easier to perform.^9^, ^10^, ^11^, ^12^ This efficiency stems from the ability to transport 2 suture threads through the meniscus with each passage using the continuous suture (CS), which streamlines the technique and reduces the time required for suture threading.

The purpose of this study was to compare the amount of time required to perform meniscal suturing on a standardized lesion using either a traditional or continuous arthroscopic suturing technique. We hypothesized that the CS method would demonstrate a faster completion time compared with the traditional method.

## Methods

### Study Design

This experimental study was conducted with the approval of the ethics committee of an educational institution (CAAE: 50899621.3.0000.5103). The study occurred at the Faculty of Medical and Health Sciences of Juiz de Fora (FCMS/JF)—SUPREMA, specifically at the Instituto Crispi Advanced Training Laboratory in Minimally Invasive Surgeries - Suprema Juiz de Fora. The training involved medical residents specializing in orthopedics and traumatology and medical doctor specialists in knee surgery. Inclusion criteria encompassed orthopedic residents and medical specialists in knee surgery. Exclusion criteria included physicians who declined to participate. All participants performed meniscal suturing using 2 distinct arthroscopic techniques to compare the time spent executing each technique.

### Participant Preparation

Preparation of the medical participants in the study was divided into 2 phases.

#### First Stage

All participants underwent online training, viewing video-format lectures demonstrating the steps of the traditional IO meniscal suture technique^13^ and the CS vertical IO meniscal repair technique^10^ before hands-on practice. On the training day, all participants attended sessions led by an experienced surgeon, conducted in 2 distinct groups (Groups 1 and 2) set up in the animal model lab. In these groups, participants received training on 2 predissected open porcine knees. In Group 1, the traditional IO suture technique was demonstrated and practiced, whereas Group 2 focused on the CS technique. Both techniques were standardized to be performed in the same manner by all participants, always starting from the most posterior point moving toward the most anterior. Once all threads had been passed through the meniscus, knots were tied using needle holders and always with the same number of knots, standardizing all participants. Every participant performed each technique at least once on these open anatomic specimens.

##### Preparation of porcine specimens

This information is detailed in Appendix 1. Porcine knee specimens were sourced from a commercial meat vendor and prepared by dissecting 15 cm around the knee, focusing on retaining the essential bony structure. These specimens were stored at temperatures ranging from –20°C to –80°C and thawed at room temperature 2 days before training. Arthroscopic access was achieved through 3 portals, and preparatory procedures including a synovectomy and pie-crust incisions in the medial collateral ligament were performed. A longitudinal lesion was created in the medial meniscus using a banana blade (Arthrex, Naples, FL). The prepared specimens were then maintained at refrigeration temperatures of 2°C to 6°C until the study commenced.

##### Data collection

The prepared porcine specimens were made available in booth 3 and 4 of the laboratory, both equipped with arthroscopy towers. Booth 3 was designated for the performance of traditional meniscal suturing, and booth 4 was designated for the performance of continuous meniscal suturing.

#### Second Stage

**Fig 1:**
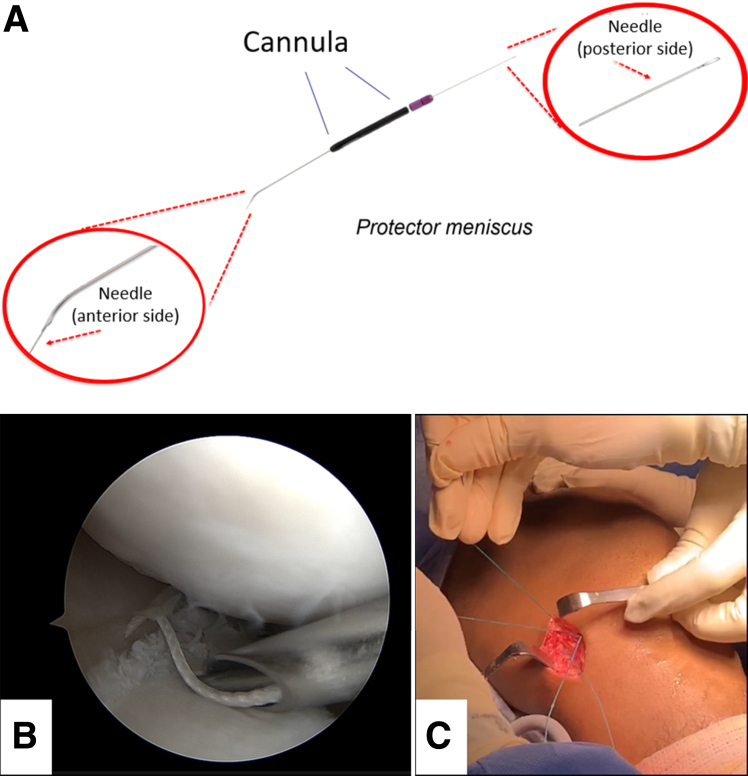
Meniscal repair using the traditional inside-out technique. (A) Meniscus protector device, with its cannula in the center, on its anterior face the end of the nitinol wire and on its posterior face the transport loop of the nitinol wire that carries the suture threads. (B) Arthroscopic view of the medial meniscus with the suture thread inserted on the proximal face of a longitudinal meniscus lesion, with the cannula being positioned for a vertical suture. (C) View of the posteromedial surgical access with the suture threads that have already been transported.

**Fig 2:**
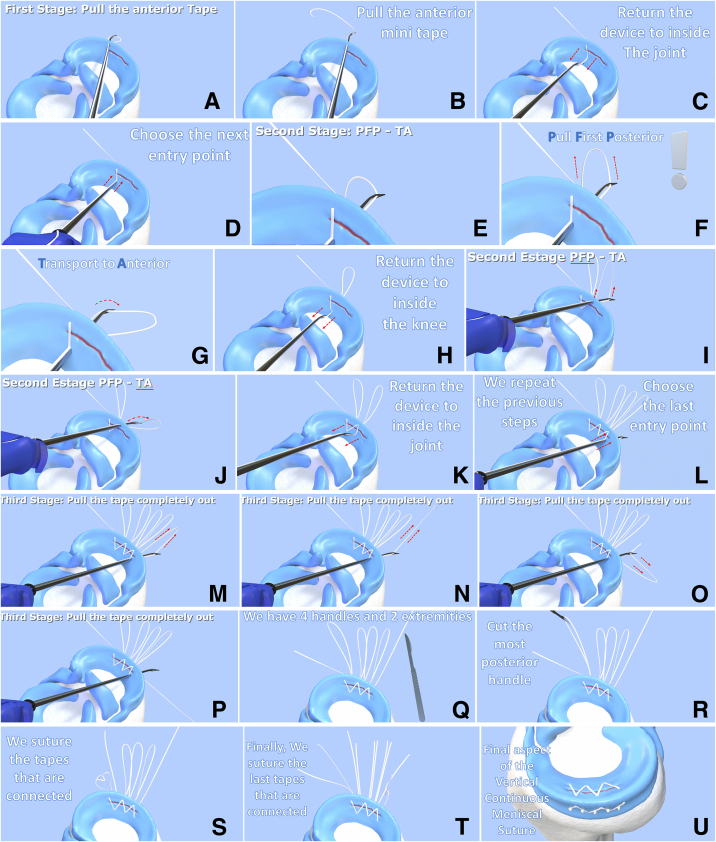
Meniscal repair using the vertical continuous inside-out suture technique using the Meniscus 4 AII suture device, demonstrating how to perform 5 stitches. The technique has 3 stages: Initial, middle, and final. (A and B) In the first stage, the meniscus 4-ALL is backed off slightly, forming 2 handles then pull the anterior handle out of the joint. The device is returned to the inside of the joint (C), and the next entry point is chosen (D). The second stage commences at this point. First the posterior handle is pulled (E and F), the handle is transported to the anterior face of the device (G). This is referred to as pull first posterior—transport to anterior (PFP-TA). (H) The Meniscus 4-ALL is returned to the inside of the knee. (I–L) The second stage is repeated as many times as necessary. (L) The last entry point is chosen. (M–P) Then the third and last stage commences, with the mini-tape pulled completely out of the joint and device. (Q) In this case, there are 4 handles and 2 tape extremities of the tape. (R) The most posterior handle is cut. (S) The tapes that are connected are sutured. (T) Finally, the last tapes that are connected are sutured. (U) This panel shows the final aspect of the vertical continuous meniscal suture. In the case shown here, we illustrate 5 points made with the continuous vertical inside-out suture. In the current study, 4 continuous points were made, which is 1 fewer than illustrated here. Adapted from Rocha de Faria (2020).^10^

For a detailed description of this stage, see Appendix 1. In this study, we performed the arthroscopic training using a porcine knee model. Two meniscal suture techniques were used: traditional IO suturing using the Protector Meniscus suturing device (Arthrex) (Fig 1) and continuous vertical suturing using the Meniscus 4 AII device (Síntegra Surgical Sciences, Pompéia, Brazil) (Fig 2), each performed in dedicated booths. Participants, randomized to the start technique, executed 4 sutures on a standardized medial meniscus tear using designated devices for each method.

Timing commenced once participants confirmed that they were ready, capturing the time required to complete 4 sutures. An assisting researcher and participant supported each performer, aiding in knee positioning and suture handling. Upon completion, technical difficulty and meniscal stability were assessed, followed by a crossover to the alternate technique.

Post-training, participants completed a questionnaire comparing their experience to human arthroscopy and the perceived benefits of continuous over traditional suturing (Table 1).

**Table 1:** Participant Characteristics

	Surgeons (n = 11)	Residents (n = 10)
Year of residency		
1	—	1 (10.00%)
2	—	4 (40.00%)
3	—	5 (50.00%)
Arthroscopies participated in		
<10	—	8 (80.00%)
10–30	—	1 (10.00%)
30–50	—	0 (00.00%)
>50	—	1 (10.00%)
Previously observed or participated in meniscal repair	—	7 (70.00%)
Time as a knee surgery specialist	7.00 (5.50, 11.50)	—
Have done any inside-out sutures	10 (90.91%)	—
Most used suture technique		
Inside-out	2 (18.18%)	—
All-inside	1 (9.09%)	—
Hybrid	8 (72.73%)	—
Outside-in	0 (00.00%)	—
No. of meniscal sutures performed monthly		
≤4	8 (72.73%)	—
>4	3 (27.27%)	—

Data displayed as n (%) for discrete data and median (the second interquartile range) for continuous variables without normal distribution.

### Statistical Analysis

In the descriptive analysis, the prior experience of orthopedic residents and surgeons was evaluated, using relative and absolute frequencies to describe the responses to the questionnaire. In the inferential analysis, the paired *t* test was used to evaluate the mean difference between the surgeons’ times for each type of suture. The assumption of normality was assessed through graphical methods.

Considering a 2-tailed alpha of 0.05 for the paired *t* test, it was determined that 21 participants would be sufficient to achieve 83% power to detect a Cohen’s *d* of ≥0.67.

Repeated-measures analysis of variance was used to evaluate the interaction of expertise with the change in suture type. All analyses were performed using R (version 4.0.5, Vienna, Austria), R Studio (version 1.4.1106), and SPSS version 26 (IBM, Armonk, NY,).

## Results

Twenty-five medical doctors were invited to participate in the study; 4 were excluded because they were unavailable on the training day, and ultimately 21 medical doctors participated. Ten anatomic specimens were used per group, along with 3 extra specimens and 2 specimens consisting only of the tibia and menisci, totaling 23 full anatomic specimens and 2 open specimens intended for prior hands-on training on the day of the study. The specimens were reused for a maximum of 3 arthroscopies to facilitate the logistics of storage and project execution without affecting practical evaluation. Three extra specimens were set aside for replacement in case any were found to be unviable during the training.

### Primary Outcome

The median [Q1–Q3] time for traditional suture was 11:57 [10:20–18:45] minutes, with an average of 14:11 minutes. The mean difference between the techniques was 4:17 minutes (4:17 minutes; 95% confidence interval: 1:46–6:46 *P* = .002), with continuous suturing being faster than the traditional technique (Table 2).

**Table 2:** Primary Analysis: Seconds and Minutes Analyzed (N = 21)

	Traditional Suture	Continuous Suture	Mean Difference	95% CI	*P* Value
Time (s)	717 (620,1125)	504 (417–698)	257 ± 330	106–406	.002
Time (min)	11:57 (10:20–18:45)	8:24 (6:57– 11:38)	4:17 ± 5:30	1:46–6:46	.002

Time required by participants in minutes				
Time	Total (N = 21)	Surgeons (n = 11)	Residents (n = 10)	*P* Value
Traditional suture (min)	11:57 (10:20–18:45)	11:01 (10:06–12:04)	17:58 (13:06–20:33)	.036
Continuous suture (min)	8:24 (6:57–11:38)	8:24 (6:59–12:40)	8:46 (6:53–10:12)	.863
P value		0.29	0.001	

Data displayed as N (%) for discrete data, median (interquartile range) for continuous variables without a normal distribution and mean ± standard deviation for continuous variables with a normal distribution. A paired *t* test was used for analysis. CI, confidence interval.

### Secondary Outcomes

Regarding the time difference between surgeons and orthopedic residents, surgeons took less time than residents for the traditional suture (*P* = .036), but the times were similar for CS (Tables 2 and 3). There was an interaction between expertise level and the type of suture used (partial η^2^ = 0.280; *P* = .014 for interaction; Table 3). A large part of the benefit of using a CS was independent of the surgeon's level of expertise (partial η^2^ = 0.484; *P* < .001; Table 3). Table 4 displays the personal impressions of the participants, the final stability of the sutures performed, and the randomized order of the suture groups.

**Table 3:** Secondary Analysis: Interaction

	η^2^ Partial	*P* Value
Suture type	0.484	<.001
Suture type × expertise	0.280	.014

A repeated-measures analysis of variance was used with the type of suture and expertise (resident/surgeon) as factors.

**Table 4:** Secondary Analyses: Participants’ Personal Impressions

	Total (N = 21) n (%)	Surgeons (n = 11) n (%)	Residents (n = 10) n (%)
How similar did you find the pig to the human knee?			
Identical	3 (14.29)	0 (0)	3 (30)
Very similar	15 (71.43)	9 (81.82)	6 (60)
Moderately similar	3 (14.29)	2 (18.18)	1 (10)
Not similar	0 (0)	0 (0)	0 (0)
Where you previously knowledgeable about the continuous suture?	11 (52.38)	7 (63.64)	4 (40)
How difficult did you find learning the continuous suture?			
Very difficult	0 (0)	0 (0)	0 (0)
Moderately difficult	2 (9.52)	0 (0)	2 (20)
A little difficult	13 (61.90)	7 (63.64)	6 (60)
No difficulty	6 (28.57)	4 (36.36)	2 (20)
Which technique did you find easier?			
Traditional inside-out suture	2 (9.52)	1 (9.09)	1 (10)
Continuous suture	19 (90.48)	10 (90.91)	9 (90)
Stability			
Traditional suture	18 (85.71)	11 (100)	7 (70)
Continuous suture	21 (100)	11 (100)	10 (100)
Order in which procedures were performed			
Continuous → traditional	15 (71.43)	7 (63.64	8 (80)
Traditional → continuous	6 (28.57)	4 (36.36)	2 (20)

## Discussion

In the present study, we observed that the CS technique applied in treating meniscal injuries was performed in less time compared with traditional suturing. Hence, the CS method was validated as a potential alternative for treating meniscal tears, offering the benefits of simplifying the treatment, reducing surgical time, and being easily reproducible.

The secondary outcomes indicate that although surgeons achieved slightly faster results (2 minutes and 37 seconds, in absolute numbers) with CS, there was no significant statistical difference between the 2 methods among surgeons. We believe that if surgeons become more familiar with the continuous vertical suture, they could potentially perform it in even less time, making the procedure quicker. However, it was observed that the continuous suture method tends to equalize the speed between surgeons and residents. Furthermore, the benefits of using continuous sutures are largely independent of the practitioner’s experience level, making it a useful technique for a wide range of health care professionals (Table 4).

An interesting indirect observation from our study was the effect of the initial randomization of participants to the suture techniques on their performance. The majority of participants (n = 15) began the study with the continuous suture technique. This initial exposure may have facilitated their adaptation to the training scenario, potentially allowing them to perform the second technique more efficiently. Specifically, it may have contributed to reduced time performing the traditional suture technique because participants had already acclimatized to the arthroscopic training and the study’s requirements by then.

Evaluating arthroscopic skills has some limitations because technical aptitude is subjective and individualistic, making it qualitative data. However, time, our chosen metric for this study, is quantitative, allowing for a more effective assessment of the technique’s efficacy.

According to Volpin et al.,^14^ the average cost per minute of operating room time is approximately £16 in the United Kingdom and $51 in the United States. Continuous meniscal suturing, compared with traditional suturing, reduces the average surgical time by 4 minutes for a 2-cm lesion treated with 4 stitches, potentially saving £64 and $204, respectively. Beyond other benefits, CS may significantly lower the final surgical costs.

In our study, when evaluating traditional suturing, there was greater variance in time between residents and specialists. Specialists took an average time of 11 minutes and 1 second, compared with residents at 17 minutes and 58 seconds. With continuous meniscal suturing, there was less disparity between specialists and residents, with respective average times of 8 minutes 24 seconds and 8 minutes 46 seconds. These findings suggest that learning and performing CS is faster than traditional methods.

Continuous meniscal suturing was described using the Meniscus4 A-II device.^10^ This device offers advantages such as rigidity and the option of straight or curved tips, facilitating repair in multiple directions and giving the surgeon better control over needle exit points. The resistance of the device also allows for its use in multiple continuous sutures, with minimal risk of breaking, potentially reducing surgical costs.

Our group published a biomechanical study that compared the same techniques evaluated in this study.^15^ We biomechanically assessed stiffness, strength, and displacement between the meniscal edges, comparing the traditional vertical IO suture group (4 stitches) with the continuous vertical IO suture group (4 stitches). We observed similar results between the 2 groups, demonstrating that the continuous vertical IO suture was biomechanically comparable to the gold standard technique in the literature.^15^^,^^16^

The vertical IO continuous meniscal suture is not indicated for lesions on the posterior or anterior horns of the meniscus. The use of this technique is limited to the transition from the body to the posterior and anterior horns.^10^ It is noteworthy that in longitudinal tears, the lesion must be repaired on both the femoral and tibial surfaces of the meniscus, which allows for greater stability and enhances the chances of meniscal healing.^17^^,^^18^

Previous studies have detailed different configurations of the CS technique, tailored to different lesion types and anatomic locations. The horizontal and vertical continuous suturing has also been described to treat bucket-handle meniscal tears,^9^^,^^10^ anterior horn lesions,^11^ and radial meniscal lesions.^12^ In bucket-handle injuries, the continuous suture technique simplifies the procedure and reduces surgical complexity. In radial meniscus tears, the method offers promising results by realigning the tear margins.

Wang et al.^19^ concluded that porcine knees are ideal for studying knee conditions and therapies due to their sufficient cartilage thickness (2–3 mm), biomechanics that closely resemble adult human conditions, and suitability for arthroscopy.

In this study, we compared 2 types of IO sutures, aiming to compare similar suture types. However, comparing the time taken to perform the continuous vertical IO suture versus the all-inside vertical suture may be an interesting evaluation for future research.

## Limitations

This study is not without limitations. The use of porcine model instead of human cadaver tissue is one limitation. However, porcine knees are a valid alternative for teaching and practicing arthroscopic techniques because of their anatomic similarity to human knees, as shown in the study by Kim et al.^20^ In our study, 81.8% of experienced surgeons found the porcine model to be very similar to the human knee.

Another limitation of the study was the use of a 100% cotton, durable thread suture thread in both techniques analyzed, which is different from that used in conventional surgeries. Also, we note the difference in skill and experience among the participants, among whom there were residents in orthopedics/traumatology and knee surgeons.

The method of using a probe to assess the stability of the lesion is subjective and also represents a limitation of the study. However, this same method is frequently used in clinical practice during meniscal repairs.

We reiterate that the continuous vertical suture has limitations in its application (e.g., with anterior and posterior horns of the meniscus). It is also important to note that ideal meniscal repair for longitudinal lesions should always be performed on both the femoral and tibial surfaces of the meniscus.^17^^,^^18^ In this study, we performed sutures only on the femoral surface to simplify the logistics of the experiment.

## Conclusions

The CS technique required less time to perform compared with the traditional suture technique in a porcine model.

## Disclosures

The authors declare the following financial interests/personal relationships which may be considered as potential competing interests: J.L.R.d.F. reports equipment, drugs, or supplies provided by Sintegra Surgical Sciences; consulting or advisory, speaking and lecture fees, and travel reimbursement from Sintegra Surgical Sciences; holds patent #US 11,589,861 licensed to Meniscal Suture Device Patent; and is junior editor of the Revista Brasileira de Ortopedia. R.F.L. reports consulting or advisory and funding grants from Smith and Nephew and Ossur Americas; funding grants from Arthrex; is on the Elsevier Committees for the American Orthopaedic Society for Sports Medicine, Arthroscopy Association of North America, International Society of Arthroscopy–Knee Surgery; and is on the editorial boards for the *American Journal of Sports Medicine*, *Knee Surgery, Sports Traumatology, Arthroscopy*, *Journal of Experimental Orthopaedics*, and *Journal of Orthopaedic & Sports Physical Therapy*. All other authors (J.L.R.F., J.V.A.N., B.C.G., D.M.P., J.A.M.G., V.M.O., R.F.L-P., D.R.R., R.S.) declare that they have no known competing financial interests or personal relationships that could have influenced the work reported in this paper.

## Supplementary Data

ICMJE author disclosure forms
