# Supplementary material for: Continuous Meniscal Repair Technique Allows for Shorter Operative Time and Learning Curve Compared With Traditional Vertical Mattress Technique in Controlled Arthroscopic Training in Porcine Model
Source: Arthrosc Sports Med Rehabil. 2024 Jun 10;6(5):100957. doi: 10.1016/j.asmr.2024.100957 (PMC11551364; doi:10.1016/j.asmr.2024.100957)
Supplement: ICMJE author disclosure forms [file mmc1.docx]

Appendix 1 – ARTH 24 – 197

CONTINUOUS MENISCAL REPAIR TECHNIQUE ALLOWS FOR SHORTER OPERATIVE TIME AND LEARNING CURVE

Material and Methods

Preparation of Porcine Specimens

The anatomical specimens were obtained from a commercial meat vendor. The porcine knee was dissected with all its osteo-myo-tendinous structure as follows: a myocutaneous dissection was performed on the porcine specimen 15 cm proximal to the knee, leaving only the bony structure in the proximal third of the femur. The tibial diaphysis was sectioned 15 cm distal to the knee, completing the initial dissection of the specimen.

The specimens were stored at temperatures between -20ºC to -80ºC and thawed at room temperature (23ºC to 25ºC) two days prior to training. On the day before the study was conducted, all the closed porcine knee specimens were accessed arthroscopically. Three arthroscopic portals were created (antero-lateral, transpatellar, and antero-medial), a broad synovectomy was performed, and "pie crust" incisions were made in the medial collateral ligament using an 18-gauge Jelco needle to expand the space in the femurotibial medial compartment. A two-centimeter-long longitudinal lesion transitioning from the red zone to the white zone in the medial meniscus body was then made using a banana blade (Arthrex Naples, FL, USA). Following this procedure, the knee specimens were refrigerated at standard refrigeration temperatures of 2 to 6 degrees Celsius until the commencement of the study.

2ND STAGE

In this study, we perform the arthroscopic training using a porcine knee model. Two different meniscal suture techniques were employed: traditional inside-out suturing (IO) and continuous vertical suturing (CS), each performed in dedicated booths. Participants, randomized to the start technique, executed four sutures on a standardized medial meniscus tear using designated devices for each method.

Timing commenced once participants were confirmed ready, capturing the duration to complete four sutures. An assisting researcher and participant supported each performer, aiding in knee positioning and suture handling. Upon finishing, technical difficulty and meniscal stability were assessed, followed by a crossover to the alternate technique.

Post-training, participants completed a questionnaire comparing their experience to human arthroscopy and the perceived benefits of continuous over traditional suturing (Table 1).

Upon initiating the arthroscopic training stage on the prepared porcine knee, traditional meniscal suturing (IO) was performed in booth 3 using the Protector Meniscus suturing device (Arthrex, Naples, FL, USA). Continuous vertical meniscal suturing (CS) was performed in booth 4, using the Meniscus 4 AII device (Síntegra Surgical Sciences, Pompéia, SP, BRA). Participants were randomly directed to the booths to begin the arthroscopic sutures. One of the authors identified the meniscus tear and positioned the meniscal suturing device in front of the meniscal lesion for each participant. Participants in booth 3 performed 4 vertical inside-out vertical mattress sutures of the body of the medial meniscus using the IO technique, as described in prior training (video and practice on an open knee) (figure 1). Participants in booth 4 performed 4 vertical mattress sutures using the CS technique, at the same meniscal location, suturing the previously prepared lesion (figure 2).

The measurement of the time participants took for each technique was carried out as follows: The participant positioned themselves as the primary surgeon in the cabin, holding the arthroscope in the lateral portal and the suture device in the medial portal. Then, one of the researchers asked the participant if they were comfortable and confident with the positioning of the knee, optics, and suture device. Upon receiving an affirmative response, the researcher inquired whether they could commence timing. Following another positive response, the timer was initiated, and the participant began performing the meniscal suture. In each cabin, a researcher and another participant observed and assisted the performing participant; the assisting participant applied knee valgus to facilitate the opening of the femorotibial space, while the researcher helped by pulling the sutures. After completing the 4 points, the timer was paused and recorded. Participants were individually assessed by the assisting researcher for technical difficulty—rated as high, medium, or low complexity—as well as for the stability of the sutured meniscus, evaluated by probing the edge of the injured meniscus at the end of the suture.

Next, the participant was taken to another cabin, and the training process for the technique not yet performed was repeated as described above. Upon completing the arthroscopic training, the participant was given a questionnaire to evaluate their experience, the degree of similarity between performing arthroscopy on a porcine knee and a human one, and also to assess any potential advantages in executing the continuous technique compared to the traditional one (Table 1).

.
